# Successful Collaboration: A Tale of a Tertiary Care Hospital and Its Designated Organ Procurement Organization

**DOI:** 10.7759/cureus.73215

**Published:** 2024-11-07

**Authors:** Keith Champlin, Robert Goodwin, Emma Miller, Crystal Yancey, Sarthak Parikh, Maryavis Howell, Rachael Ketcham, Ashley Milam, Bradon Nave, Travis Campbell, Mani Cheruvu

**Affiliations:** 1 Trauma Institute, Saint Francis Health System, Tulsa, USA; 2 LifeShare, LifeShare of Oklahoma, Tulsa, USA; 3 Graduate Medical Education and Center for Clinical Research and Sponsored Programs, Saint Francis Health System, Tulsa, USA

**Keywords:** organ donation, organ transplant, pediatrics, tissue donation, trauma

## Abstract

The death of a child is a tragedy of astronomical proportions. Pediatric trauma patients often have injuries so extensive that there is no hope of recovery. Though the tragedy can never be undone, organ donation can save lives and create a legacy for the donor and their loved ones. Organ donation is a multi-factorial and dynamic process that is further complicated in the case of pediatric trauma patients due to the sudden-onset and intense emotions experienced by the patient’s family. A critical factor in the success of organ donation is the collaborative relationship between a hospital and its respective Organ Procurement Organization (OPO). Successful organ donation relies on this relationship, as neither entity can achieve this goal on its own. By examining the relationship between Saint Francis Children’s Hospital in Tulsa, OK, and its OPO, LifeShare, this paper argues that a strong, collaborative relationship between a hospital and its OPO can maximize the rates of organ referrals and donations, thereby increasing the available supply of pediatric donors who have passed away.

## Introduction

More than 1,700 pediatric patients receive organ transplants each year [1]. Despite the apparent balance between the number of donors and transplant recipients, over 48% of pediatric patients on the waiting list (all organs) had been waiting for more than a year as of 2021 [2]. Given the increasing demand for transplantable organs, it is now more critical than ever to contribute to the literature on pediatric organ donation [3]. Notably, children, particularly those under the age of 5, face the highest mortality rates on the transplant waiting list (all organs) compared to any other age group [4].

In addition to the rising number of pediatric patients in critical need of new organs, a recent survey conducted in the United Kingdom, Spain, and the USA revealed that overall pediatric donation rates are significantly lower than those for adults. Furthermore, most organs recovered from pediatric donors were allocated to adult recipients [5].

Pediatric transplant trends in Oklahoma mirrored national patterns from 2018 to 2022. Both nationally and in Oklahoma, pediatric donor rates increased until 2020, when a sharp decline was observed, likely due to the COVID-19 pandemic. Following 2020, the nationwide number of pediatric donors rebounded, aligning with pre-pandemic trends, while the number in Oklahoma remained relatively stagnant.

Although Oklahoma's donor trends have been similar to national trends, recipient trends have diverged. During this period, transplant centers in Oklahoma only performed intra-abdominal organ transplants, whereas nationally, programs were transplanting both thoracic and intra-abdominal organs for pediatric patients. Nationwide, the number of pediatric transplant recipients decreased from 1,895 in 2018 to 1,777 in 2022, while in Oklahoma, the number of pediatric transplant recipients increased during the same period. In 2020, the impact of COVID-19 is evident; there was a nationwide decrease in pediatric transplant recipients compared to other years reviewed in this study. Interestingly, however, Oklahoma saw an increase in the number of pediatric patients receiving life-saving organ transplants in 2020.

This study aims to review how the evolution of the relationship and degree of collaboration between Saint Francis Children’s Hospital (SFCH) in Tulsa, OK, and its organ procurement organization (OPO), LifeShare, has impacted the success rate of the hospital’s organ and tissue donation program. A strong, collaborative relationship between a hospital and its OPO can maximize the rates of organ and tissue referrals and donations, thereby increasing the available supply of pediatric donors who have passed away. The tangible efforts made to improve relationships and collaboration between these two entities will be examined.

## Materials and methods

This retrospective study used data obtained from Saint Francis Children’s Hospital’s (SFCH) and LifeShare Oklahoma’s (OPO) electronic medical records (EMR) between 2018 and 2022. All organ and tissue referrals made by SFCH and Saint Francis Hospital to the OPO during this time were reviewed. Adult data were also reviewed at aggregated levels to help measure the potential impacts of hospital-wide collaborative efforts since the pediatric donation referral population is so small. From this larger dataset, all pediatric organ donors under 15 years of age who died by way of trauma injury received additional attention and analysis. Forty-seven pediatric trauma patients donated organs during the study period. After excluding all patients over 15 years of age, 18 cases remained. Demographic data, including sex, age, height, weight, BMI, and blood type, were collected. Once this cohort was identified, additional data were gathered from LifeShare Oklahoma’s EMR, including the donor’s cause of death and the number of organs procured, transplanted, donated to research, and discarded. Key performance metrics provided by the OPO related to referrals and donors are used to assess the overall success of a hospital’s organ and tissue donation program. Performance metrics examined include the percentage of donation conversations with potential donor families that follow hospital policy and plan as established in coordination with LifeShare (planned approach rate), the percentage of potential donors who became donors (potential rate), and the percentage of expected donors according to the United Network for Organ Sharing eligibility criteria who become donors (conversion rate).

## Results

Over the five-year period from 2018 to 2022, organ and tissue donation activity at Saint Francis Health System demonstrated fluctuating trends across several key metrics for both pediatric and adult patients.

Organ referrals showed variability, with pediatric referrals peaking at 39 in both 2019 and 2021, while adult referrals increased significantly from 551 in 2018 to a peak of 1,019 in 2021, followed by a decline to 813 in 2022. In total, pediatric referrals were recorded 32 in 2022.

The number of organ donors also varied, with pediatric donors reaching a high of 10 in both 2019 and 2022, while adult donors increased steadily from 35 in 2018 to 55 in 2022.

Organs recovered exhibited significant fluctuations, with the highest recovery count of 40 organs for pediatrics in both 2018 and 2022 and an increase in adult organ recoveries from 135 in 2018 to 214 in 2022. The recovery of organs for research ranged from 2 to 7 for pediatric patients, with 6 noted in both 2019 and 2022, while adult organs for research rose markedly from 14 in 2018 to 56 in 2022.

A total of three organs were discarded in 2022 for pediatric cases, while no organs were discarded in 2018 and 2021. The organs transplanted followed a similar trend, with the highest number of 33 pediatric transplants in 2018 and a low of 8 in 2020; adult transplants peaked at 151 in 2022.

Pediatric lives saved fluctuated, with a peak of eight lives saved in 2018 and a low of two lives saved in both 2020 and 2021, improving to five lives saved in 2022. The total number of lives saved, including adults, declined to seven in 2020 but rose to 130 by 2022.

**Figure 1:**
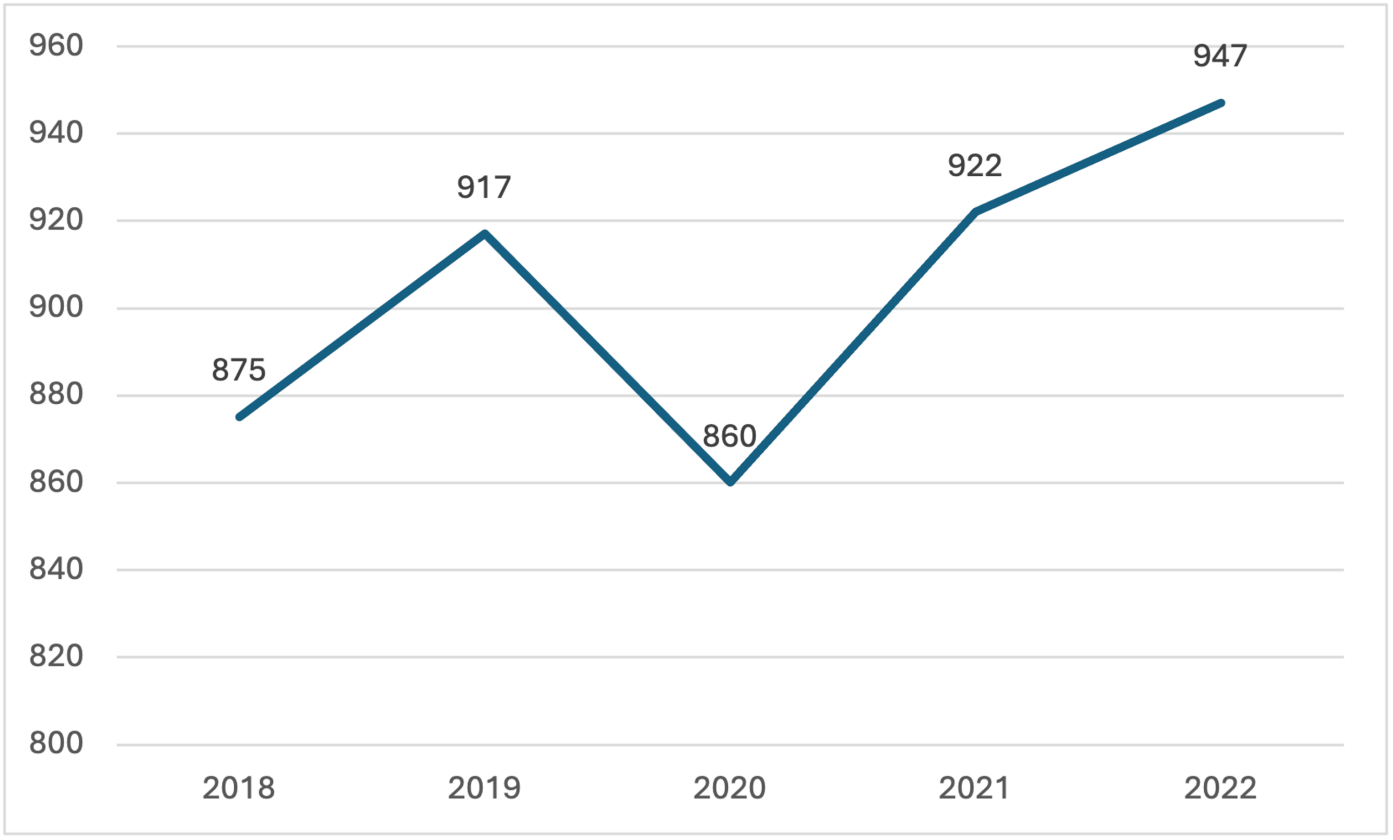
Pediatric Donors Recovered in the United States The data has been represented as N = number of pediatric donors by year

**Figure 2:**
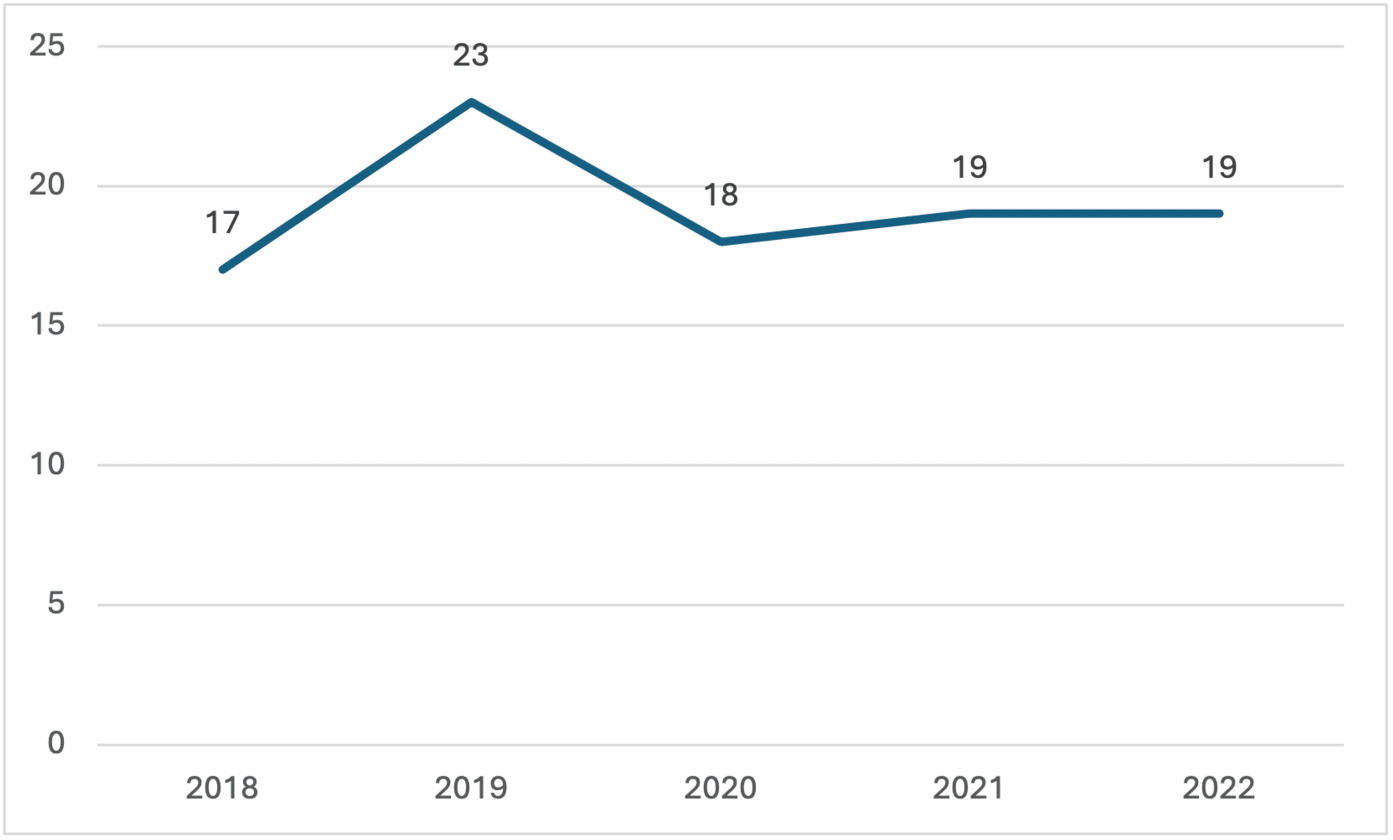
Pediatric Donors Recovered in Oklahoma The data has been represented as N = number of pediatric donors by year

**Figure 3:**
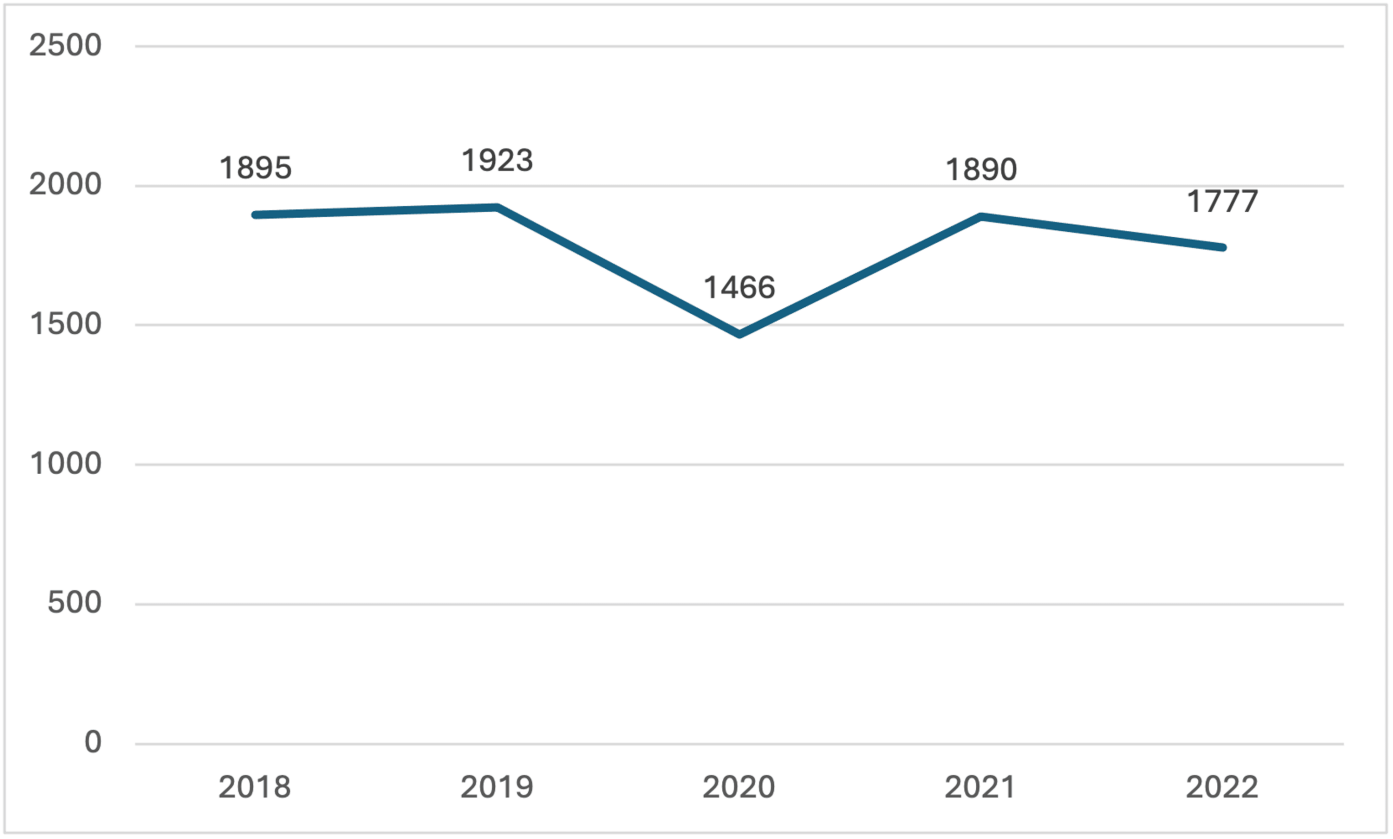
Pediatric Transplant Recipients in the United States The data has been represented as N = number of pediatric transplant recipients by year

**Figure 4:**
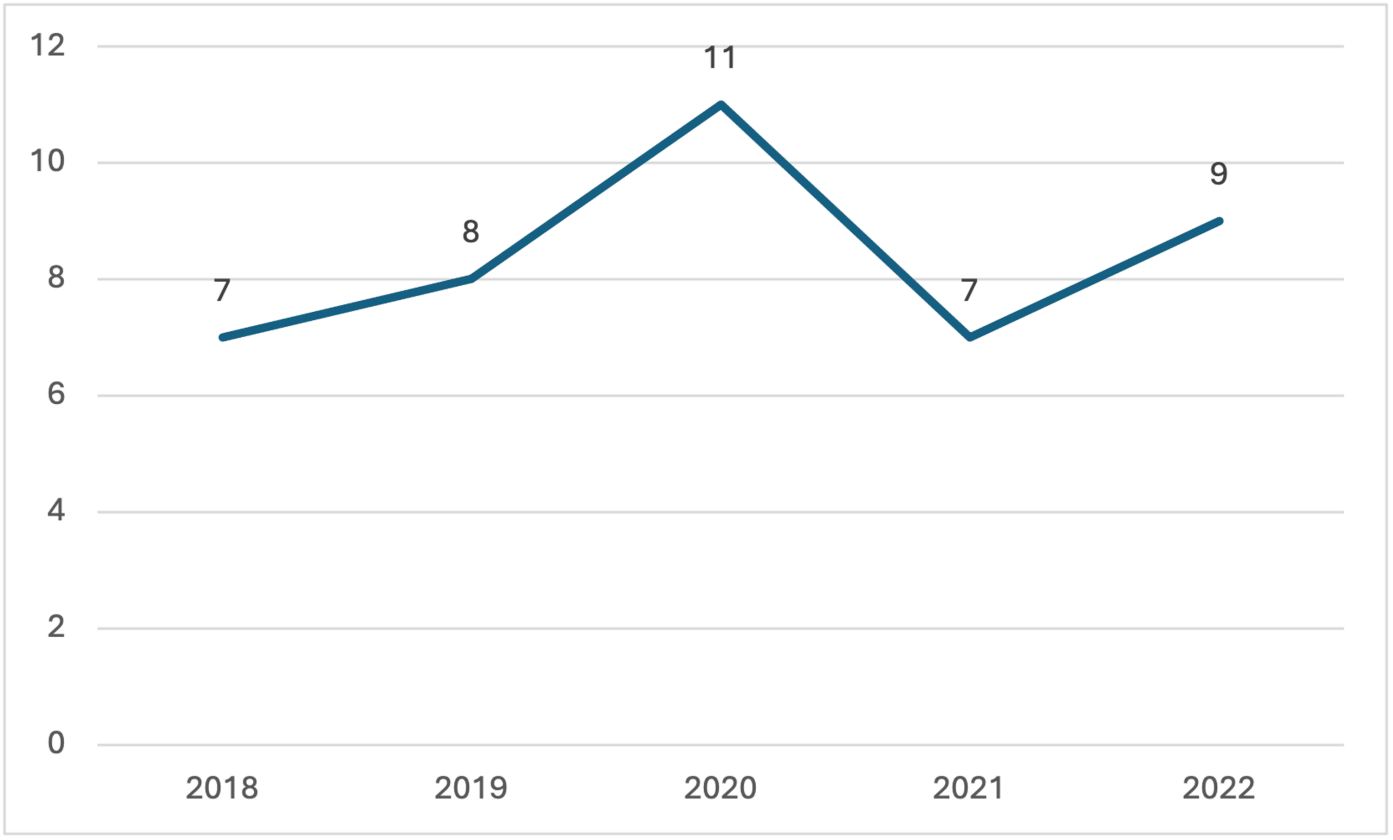
Pediatric Transplant Recipients in Oklahoma The data has been represented as N = number of pediatric transplant recipients by year

Tissue referrals showed an increase, particularly in 2021 with 97 pediatric referrals, while adult tissue referrals increased dramatically, particularly in 2020 with 2,071 referrals and remained substantial at 2,128 in 2022. The number of tissue donors exhibited variation but ultimately increased to 120 in 2022 from 93 in 2018 (Table 1) (Figures 1-4).

**Table 1:** Organ and Tissue Donation Activity at Saint Francis Health System (2018-2022) Adult (Pediatric): The first number represents the total for adults, while the number in parentheses indicates the pediatric figures. Percentages: Values in parentheses after the pediatric numbers show the percentage of the total for that category. N=number of donors

Year	Organ Referrals	Organ Donors	Organs Recovered	Organs for Research	Organs Discarded	Organs Transplanted	Lives Saved	Tissue Referrals	Tissue Donors
	Adult (Pediatric)	Adult (Pediatric)	Adult (Pediatric)	Adult (Pediatric)	Adult (Pediatric)	Adult (Pediatric)	Adult (Pediatric)	Adult (Pediatric)	Adult (Pediatric)
2018	551 (28, 5.1%)	35 (9, 20.0%)	135 (40, 29.6%)	14 (7, 33.3%)	9 (0, 0.0%)	114 (33, 29.0%)	101 (8, 7.9%)	907 (60, 6.6%)	93 (7, 7.0%)
2019	694 (39, 5.6%)	44 (10, 22.7%)	156 (37, 23.7%)	19, (6, 31.6%)	28 (4, 14.3%)	117 (27, 23.1%)	102 (4, 3.9%)	1044 (54, 5.2%)	85 (6, 7.1%)
2020	809 (22, 2.7%)	42 (5, 11.9%)	147 (14, 9.5%)	19 (2, 10.5%)	29 (4, 13.8%)	104 (8, 7.7%)	90 (2, 2.2%)	2071 (73, 3.5%)	110 (6, 5.5%)
2021	1019 (39, 3.8%)	51 (7, 13.7%)	193 (29, 15.0%)	37 (7, 18.9%)	30 (0, 0.0%)	142 (22, 15.5%)	122 (2, 1.6%)	2280 (97, 4.3%)	95 (6, 6.3%)
2022	813 (32, 3.9%)	55 (10, 18.2%)	214 (40, 18.7%)	56 (6, 10.7%)	32 (3, 9.4%)	151 (31, 20.5%)	130 (5, 3.8%)	2128 (92, 4.3%)	120 (9, 7.5%)

## Discussion

Saint Francis Hospital (SFH) and LifeShare of Oklahoma have taken deliberate steps to enhance collaboration and create a positive environment for organ and tissue donation. Before 2018, SFH faced challenges such as being understaffed and high turnover among the LifeShare staff assigned to support the hospital. At that time, only two LifeShare coordinators were assigned to the hospital, leading to frequent cancellations of educational sessions on organ donation due to insufficient staff. Additionally, LifeShare was removed from the orientation process for new critical care nurses by the Donation Council.

Recognizing the need for improvement SFH has since made concerted efforts to better integrate the LifeShare team, providing numerous opportunities for engagement at every level [6-11]. Beginning in 2019, LifeShare personnel were included in emergency department and pediatric nursing orientations. Regular quarterly donation committee meetings began in April 2019, and donation activities were incorporated into the hospital's annual quality assurance and performance improvement reviews. LifeShare nurses are now included in new nurse orientations, and regular educational sessions are provided to critical care teams.

Since the fourth quarter of 2020, LifeShare has designated an employee to work on-site at SFH at least twice a week during night shifts, with additional presence as needed for active donation cases. By April 2021, three LifeShare employees were specifically assigned to support all donation efforts at SFH. The hospital even provided office space with the Trauma Services Team for these coordinators [12]. This daily rounding through critical care areas allows for face-to-face interaction, relationship-building, and real-time support for potential donor referrals, which is crucial for pediatric organ donation and transplantation. Most parents and families have not considered the possibility of organ donation for their child, and primary care physicians typically do not discuss this during well-child visits. According to the Centers for Medicare and Medicaid Services Conditions of Participation, the OPO must be involved in discussions about organ donation as soon as a child meets the referral criteria [13]. Research indicates that limiting these conversations to end-of-life care scenarios results in missed opportunities to educate and support families who might be open to exploring donation before their child faces a critical illness [2,14].

Before 2018, the hospital’s informatics team worked with LifeShare to grant EMR access to LifeShare coordinators, enabling them to add communication notes to donors' charts. Since 2018, the informatics team has supported multiple process improvement projects related to donations. In August 2020, in collaboration with the pharmacy and pediatric multidisciplinary team, LifeShare established pediatric-specific dosing guidelines for organ donors, the first of their kind in Oklahoma [15]. This initiative has had a positive impact state-wide, benefiting every pediatric donor cared for by the OPO.

In September 2022, LifeShare was integrated into the Trauma Team’s process improvement meetings, and the Donation Council now holds regular meetings with active participation from both SFCH and LifeShare. Since 2019, the physician group has received annual education on organ and tissue referral and donation processes from various medical teams, including hospitalists, critical care, trauma, stroke teams, and pediatric intensivists. Grand rounds to enhance knowledge and awareness of the physician's role in donation began in 2021, and a section on donation was added to the new physicians’ onboarding handbook in July 2019.

A joint effort in January 2021 led to the implementation of a process for securing a pronouncing physician for donation after circulatory death (DCD) donors. This process involves LifeShare contacting the executive director, who then notifies the hospitalists about the date and time of withdrawal for a DCD donor, allowing them to volunteer as the pronouncing physician. SFH’s growth in donor numbers from 2018 to 2022 is largely attributable to an increase in DCD donors. In 2018, there were 35 organ donors, 4 (11%) of whom were DCD donors; by 2022, the number of donors had risen to 55, with 23 (42%) being DCD donors, a 57% increase in donors overall, with a six-fold increase in DCD donors.

In April 2021, the informatics team supported another process improvement by adding a “Dr. LifeShare” option in the EMR to be selected as the attending physician following brain death pronouncement, along with an integrated power plan order set. In September 2022, the team optimized the postmortem flow sheet in the EMR by separating key questions to reduce confusion among nursing staff about calling in tissue referrals after an organ referral, in line with U.S. Centers for Medicare & Medicaid Services guidelines. SFH will also be the first hospital in the state to implement auto-referrals to LifeShare from within the EMR, further streamlining the referral process and saving valuable time for the nursing team. These collaborative process improvement initiatives foster trust-filled relationships, build confidence in our collective ability to save lives through donation, and enable SFH to maintain a successful organ and tissue donation program.

A 2016 systematic review [16] evaluated 23 original articles on "indicators of efficiency" in the organ donation and transplantation process. These indicators were categorized into three main areas: organ donation, organ transplantation, and patient demand/hospital resources. Donation efficiency indicators include mortality statistics, communication of brain death, clinical status of donors, exclusion of donors for medical reasons, family attitudes, confirmation of donations, and the extraction of organs and tissues. Transplantation efficiency indicators involve aspects of the surgical procedure and post-transplantation follow-up, while the final group considers the overall climate of transplantation for both patients and the hospital. The more indicators included in a process, the more efficient that process is. Based on these indicators, the organ donation and transplantation process executed by the SFCH and LifeShare partnership is highly efficient, though clear areas for improvement remain.

While this study highlights the importance of a strong collaborative relationship between SFCH and LifeShare Oklahoma in maximizing organ donation rates, it is important to acknowledge several limitations. First, the study's retrospective design and reliance on data from a single hospital and OPO may limit the generalizability of the findings to other institutions or regions. Additionally, the small sample size of pediatric donors, particularly those under 15 years of age, constrains the ability to draw broader conclusions about pediatric organ donation trends. The study also lacks a comprehensive analysis of external factors, such as socioeconomic status, cultural differences, and family dynamics, which may significantly impact donation decisions. Furthermore, the potential influence of the COVID-19 pandemic on organ donation and transplantation trends, while mentioned, is not fully explored, leaving some uncertainties regarding the broader implications of the findings. Finally, the study does not compare the outcomes of the SFH and LifeShare collaboration with those of other hospital-OPO partnerships, which could provide a more robust understanding of best practices in this field.

## Conclusions

Successful organ donation relies on the strength of the relationship between a hospital and its OPO. Organ referrals and donations are maximized when that relationship is strengthened, resulting in more lives saved. This study underscores the critical role of collaboration, communication, and shared goals between Saint Francis Children’s Hospital and LifeShare Oklahoma in enhancing pediatric organ donation outcomes. By integrating OPO personnel into hospital processes, fostering continuous education, and implementing targeted process improvements, both entities have created a framework that supports a higher conversion rate of potential donors to actual donors. Although challenges remain, particularly in the areas of sustaining these gains amidst external pressures like the COVID-19 pandemic, the experience at Saint Francis serves as a model for how dedicated partnerships can significantly impact the success of organ donation programs. Continued focus on refining these collaborative efforts and addressing identified limitations will be crucial in further improving donation rates and saving more lives.
